# Acute kidney injury: a strong risk factor for hypoglycaemia in hospitalized patients with type 2 diabetes

**DOI:** 10.1007/s00592-023-02112-0

**Published:** 2023-05-13

**Authors:** Ana Carreira, Pedro Castro, Filipe Mira, Miguel Melo, Pedro Ribeiro, Lèlita Santos

**Affiliations:** 1 Department of Endocrinology, Diabetes and Metabolism, Centro Hospitalar E Universitário de Coimbra, 3004-561 Coimbra, Portugal; 2 Department of Nephrology, Centro Hospitalar E Universitário de Coimbra, 3004-561 Coimbra, Portugal; 3 Department of Internal Medicine, Centro Hospitalar E Universitário de Coimbra, 3004-561 Coimbra, Portugal; 4 Faculty of Medicine, Coimbra University, Coimbra, Portugal

**Keywords:** Hypoglycemia, Acute kidney injury, Type 2 diabetes, Hospitalization

## Abstract

**Aims:**

Acute kidney injury (AKI) is highly prevalent during hospitalization of patients with type 2 diabetes (T2D). We aimed to assess the impact of AKI and its severity and duration on the risk of hypoglycaemia in hospitalized patients with T2D.

**Methods:**

Retrospective cohort analysis of patients with T2D, admitted at a University Hospital in 2018–2019. AKI was defined as an increase in serum creatinine by ≥ 0.3 mg/dl (48 h) or ≥ 1.5 times baseline (7 days), and hypoglycaemia as blood glucose concentration < 70 mg/dl. Patients with chronic kidney disease stage ≥ 4 were excluded. We registered 239 hospitalizations with AKI and randomly selected 239 without AKI (control). Multiple logistic regression was used to adjust for confounding factors and ROC curve analysis to determine a cutoff for AKI duration.

**Results:**

The risk of hypoglycaemia was higher in the AKI group (crude OR 3.6, 95%CI 1.8–9.6), even after adjusting for covariates (OR 4.2, 95%CI 1.8–9.6). Each day of AKI duration was associated with a 14% increase in the risk of hypoglycaemia (95%CI 1.1–1.2), and a cutoff of 5.5 days of AKI duration was obtained for increased risk of hypoglycaemia and mortality. AKI severity was also associated with mortality, but showed no significant association with hypoglycaemia. Patients with hypoglycaemia had 4.4 times greater risk of mortality (95%CI 2.4–8.2).

**Conclusions:**

AKI increased the risk of hypoglycaemia during hospitalization of patients with T2D, and its duration was the main risk factor. These results highlight the need for specific protocols to avoid hypoglycaemia and its burden in patients with AKI.

## Introduction

Hypoglycaemia is common during hospitalization of any cause in patients with diabetes and has been associated with increased length of stay and mortality [1–5]. Whether hypoglycaemia is a direct cause for the increased mortality or a marker of severe disease is still a matter of debate [6, 7]. Nonetheless, increasing evidence suggests a bidirectional association between severe hypoglycaemia and cardiovascular events in type 2 diabetes, possibly explained by a common at-risk phenotype of frail patients [8, 9]. Hence, older age, lower weight, longer diabetes duration, lower glomerular filtration rate (GFR) and therapies including insulin or sulfonylurea confer greater risk for hypoglycaemia and warrant special considerations about insulin dosing during hospitalization [3, 4, 8, 9]. Chronic kidney disease (CKD), particularly in advanced stages (4–5), is a well-recognized risk factor for hypoglycaemia, due to decreased renal clearance of insulin and other hypoglycaemic medications, decreased renal gluconeogenesis, increased insulin resistance and impaired counterregulatory hormone response [10, 11]. Therefore, a reduction in insulin dose is often recommended in these patients. Large modifications of kidney metabolism, including impaired gluconeogenesis, also occur during acute kidney injury (AKI), but their impact on systemic metabolism is less clear [12]. AKI occurs in 1 in every 5 adults hospitalized worldwide [13], with higher frequency in patients with diabetes, leading to increased mortality [14]. Recent animal experiments suggest that AKI might be a major determinant of systemic glucose levels in conditions of acute stress [12], and studies of patients in intensive care units (ICU) reported an up to tenfold greater risk for hypoglycaemia in patients with AKI [15]. Nonetheless, these studies were performed under intensive insulin therapy protocols, with demanding glucose targets, that also contributed for the risk of hypoglycaemia [15, 16]. In fact, most hospitalized patients with type 2 diabetes are admitted in general medical wards, but studies addressing the impact of AKI on the risk of hypoglycaemia are scarce outside the ICU setting. Recently, a retrospective study demonstrated that patients with AKI during hospitalization of any kind had increased risk for hypoglycaemia after hospital discharge, and that the risk was greater for patients with lesser degrees of renal recovery at discharge [17]. Nevertheless, the risk for hypoglycaemia in patients with diabetes that experience AKI while hospitalized in general medical wards is still undefined and adjusted protocols for insulin dosing during hospitalization and after discharge are lacking.

Furthermore, to our knowledge, the impact of AKI severity and duration on hypoglycaemia risk during hospitalization has not been clarified yet.

Therefore, in this study, we hypothesized that AKI increases the risk for hypoglycaemia during hospitalization of patients with type 2 diabetes in general medical wards. Simultaneously we aimed to assess the association between the severity and duration of AKI and the risk for hypoglycaemia.

## Subjects, materials and methods

### Study design and participants

We conducted a retrospective cohort study comprised of adult patients with type 2 diabetes, hospitalized in internal medicine wards at the Coimbra Hospital and University Centre, Portugal, from January 1, 2018, to December 31, 2019. Data were collected from electronic health records and discharge letters. A total of 1881 patients had a diagnosis of diabetes according to ICD-10 codes E10–E14. The exposure of interest was AKI and the outcome of interest was hypoglycaemia during hospitalization. Patients with advanced CKD (stages 4–5), without baseline ambulatory creatinine value in the previous 12 months, with diabetes classification other than type 2, or without capillary glycemia measurements during hospitalization were excluded. Patients with diagnosed AKI that did not meet our AKI definition criteria were also excluded. Patients with AKI were selected to form the exposure group and an equal number of patients without AKI were randomly selected to form the control group. Extracted data included patient demographics, comorbidities, medication, laboratory test results, daily capillary glycemia monitoring, length of stay and date of death. Estimated GFR (eGFR) was calculated using the CKD-EPI equation. This study was approved by the ethics committee of Coimbra Hospital and University Centre (reference CHUC-148-20).

### AKI definition

AKI was defined according to the Kidney Disease: Improving Global Outcomes (KDIGO) guidelines, by an increase in serum creatinine (SC) by ≥ 0.3 mg/dl within 48 h or an increase in SC ≥ 1.5 times baseline within 7 days. Urine output was not used to define AKI since most records were inaccurate and would not portray a reliable source of results. Severity of AKI was calculated as the ratio between maximum SC during hospitalization and baseline SC, and staged according to KDIGO recommendations, with stage 2 comprising increases in SC of 2–2.9 times baseline and stage 3 of ≥ 3 times baseline or ≥ 4 mg/dl and/or need for hemodialysis [14]. SC laboratory data were extracted for this purpose. Baseline creatinine was defined as the previous ambulatory creatinine value in the 12 months before admission. AKI duration was calculated as the number of days between AKI identification and the return of SC to values ≤ 0.3 mg/dl above baseline or ≤ 1.5 times baseline, respectively according to the criteria applied at first.

### Outcomes

The primary outcome was hypoglycaemia. Hypoglycaemia was defined as a blood glucose concentration < 70 mg/dl, according to the American Diabetes Association's Standards of Medical Care in Diabetes. It was classified as mild if blood glucose was < 70 mg/dl and ≥ 54 mg/dl (level 1) and as severe if blood glucose < 54 mg/dl (level 2) or if altered mental and/or physical status, requiring assistance for treatment (level 3) [18]. Hypoglycaemic events were evaluated and quantified by using capillary blood glucose records, with approximately 4–7 records per day, and laboratory glucose values. Daily medical records were examined for evidence of altered mental and/or physical status during hypoglycaemia. Time between AKI and hypoglycaemia was calculated as the number of days between AKI identification and the first hypoglycaemic event. Hypoglycaemia occurring before AKI identification was also registered.

### Statistical analysis

Analyses were performed with the use of IBM SPSS Statistics 26.0. Categorical variables are presented as frequencies and percentages, and continuous variables as means and standard deviations, or medians and interquartile ranges for variables with skewed distributions. The means or medians of continuous variables were compared between patient groups using the Student's T-test for independent samples or the Mann Whitney test, respectively. Associations between categorical variables were assessed using the Chi-square test. Multiple logistic regression was used to control for sample heterogeneity and possible confounding factors. Independent variables included the exposure of interest and potential confounders (age, estimated glomerular filtration rate, institutionalization status, hypertension, prior insulin therapy, primary diagnosis of infectious disease, insulin dose reduction on admission and insulin regimen during hospitalization), that were selected from bivariate analysis (*p* < 0.1). ROC curve analysis and Youden index were used to determine specific cutoff values for AKI duration. All reported *P* values are two-tailed, with a *P* value of less than 0.05 indicating statistical significance.

## Results

### AKI and hypoglycaemia

From the total of 1881 hospitalized patients with diabetes, 436 patients (23.2%) had a diagnosis of AKI (ICD-10: N17) during hospitalization. After applying the exclusion criteria, we obtained a total of 239 patients with AKI. The same number (*N* = 239) of patients without AKI was randomly selected, constituting a global sample of 478 hospitalized patients with type 2 diabetes. Baseline characteristics of both groups are presented in Table 1.

**Table 1:** Baseline characteristics of the patients

Characteristic	With AKI (*N* = 239)	Without AKI (*N* = 239)	*P* value
**Demographics**			
Age (years), mean ± SD	82.7 ± 7.9	80.3 ± 10.1	0.004
Female sex, n (%)	152 (63.3)	135 (56.5)	0.112
Living in nursing home or health care facilities, n (%)	89 (37.2)	70 (29.3)	0.065
**Laboratory values**			
HbA1c (%), mean ± SD*	7.5 ± 1.8	7.3 ± 1.4	0.268
eGFR (ml/min/1.73 m^2^), mean ± SD	59.0 ± 17.3	70.7 ± 19.1	< 0.001
**Previous diabetes therapy**			
Insulin, n (%)	95 (39.7)	77 (32.2)	0.086
Basal only	81 (82.7)	58 (76.3)	0.301
TDID*			0.292
0–20U	36 (38.7)	39 (50.6)	0.119
21–40U	34 (36.6)	22 (28.6)	0.270
> 40U	23 (24.7)	16 (20.8)	0.542
Other antihyperglycaemic medications, n (%)	167 (69.9)	167 (69.9)	0.540
Including sulfonylurea	14 (5.9)	15 (6.3)	0.857
**Chronic conditions**			
Hypertension, n (%)	214 (89.5)	187 (78.2)	0.001
Dyslipidaemia, n (%)	126 (52.7)	131 (54.8)	0.646
Chronic kidney disease (stages 1–3), n (%)	147 (61.5)	106 (44.4)	< 0.001
**Hospitalization**			
Primary diagnosis of infectious disease, n (%)	164 (68.6)	142 (59.4)	0.036
Insulin regimen, n (%)			< 0.001
Sliding scale	63 (26.4)	89 (37.2)	0.011
Basal only	36 (15.1)	12 (5.0)	< 0.001
Basal and bolus	140 (58.6)	138 (57.7)	0.853
TDID reduction at D0, n (%)*	66 (61.7)	34 (44.7)	< 0.001
Duration (days), median (IQR)	10 (7–16)	10 (8–14)	0.433

*IQR* Interquartile Range (Q1-Q3); *SD* standard deviation; *eGFR* estimated Glomerular Filtration Rate; *TDID* Total Daily Insulin Dose; *D0* Day 0 (immediately at admission)

*71 missing values for HbA1c; 2 missing values for Total Daily Dosage; 4 missing values for TDID reduction at D0

Patients with AKI were significantly older (82.7 ± 7.9 vs 80.3 ± 10.1 years, *p* = 0.004), had lower baseline eGFR (59.0 ± 17.3 vs 70.7 ± 19.1 ml/min/1.73 m^2^, *p* < 0.001), higher prevalence of hypertension (89.5 vs 78.2%, *p* = 0.001) and were more frequently admitted for infectious diseases (68.6 vs 59.4%, *p* = 0.036). There was no significant difference in baseline glycaemic control, assessed by glycated hemoglobin. The most frequent etiology of AKI was prerenal (88.3%), followed by renal (8.4%) and post-renal (3.3%). AKI median duration was 4 days (interquartile range [IQR]) 2–7 days), and AKI severity ranged from stage 1 (52.3%) to stage 2 (31.0%) and 3 (16.7%). Hypoglycaemia during hospitalization occurred in 97 (40.6%) patients in the AKI group versus 38 (15.9%) patients in the control group. Additionally, 48 (20.1%) patients with AKI and 22 (9.2%) patients without AKI had at least one event of severe hypoglycaemia. Analyzing the patients in the AKI group who had hypoglycaemia, 93 (95.9%) experienced the first hypoglycaemic event after AKI identification, with a median delay of 6 days (IQR 4–9 days). As displayed in Table 2, the risk of hypoglycaemia was higher in patients in the AKI group, and this association remained significant after adjustment for covariates, with 4.2 times greater odd for hypoglycaemia (95% CI: 1.8–9.6) in patients with AKI during hospitalization. In addition, there was also a trend toward a higher risk of severe hypoglycaemia in the AKI group.

**Table 2:** Multiple logistic regression model to compare hypoglycaemia (A) and severe hypoglycaemia (B) between groups

Variable	Crude OR	95% CI	*p* value	Adjusted^a^ OR	95% CI	*p* value
*A. Hypoglycaemia*						
AKI	3.6	2.3–5.6	< 0.001	4.2	1.8–9.6	0.001
*B. Severe hypoglycaemia*						
AKI	2.5	1.4–4.3	0.001	2.5	1.0–6.4	0.053

*CI* Confidence Interval; *OR* Odds Ratio

^a^Adjusted for: age, estimated glomerular filtration rate, institutionalization status, hypertension, prior insulin therapy, primary diagnosis of infectious disease, insulin dose reduction on admission and insulin regimen during hospitalization

**Fig. 1:**
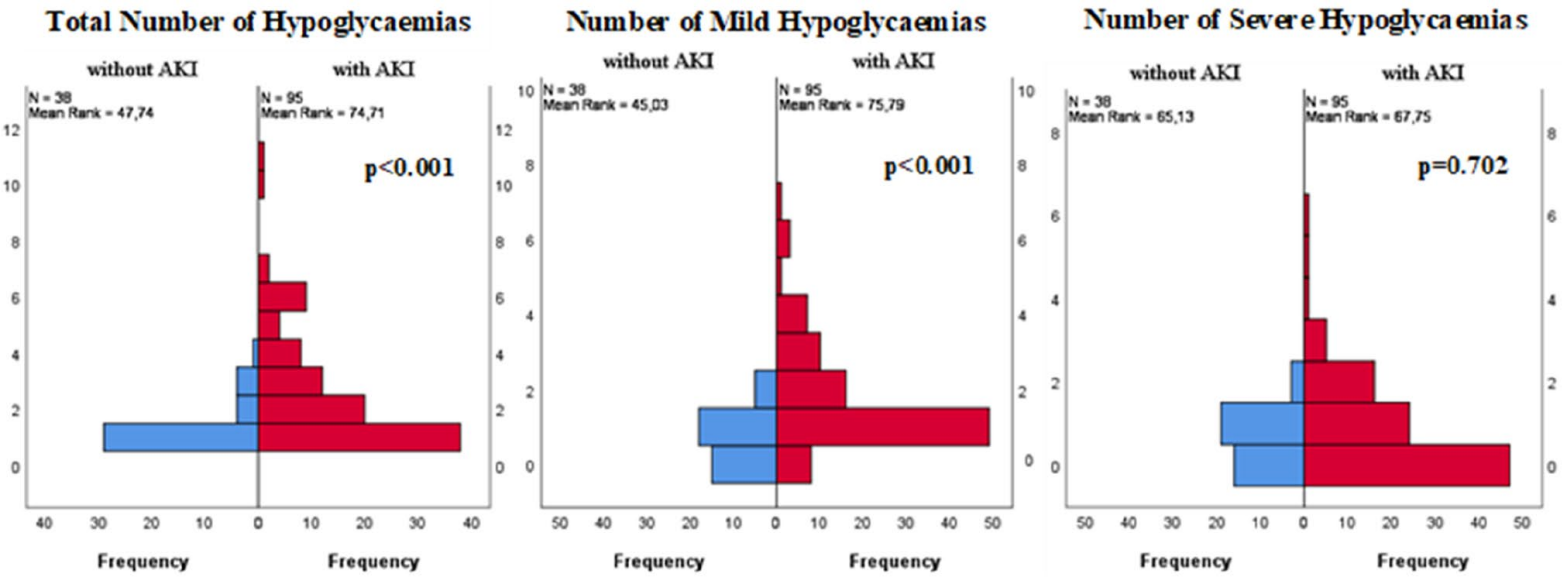
Comparison of the number of total, mild and severe hypoglycaemias between groups. Independent-Samples Mann–Whitney U Test, according to group type. Only hypoglycaemias that occurred after AKI were counted in the AKI group. Patients who had only hypoglycaemia prior to AKI were excluded (*N* = 2)

Furthermore, comparing the patients with hypoglycaemia, those in the AKI group had a higher median number of hypoglycaemic events during hospitalization than those without AKI (2.0 [IQR 1.0–4.0] vs. 1.0 [IQR 1.0–1.3], *p* < 0.001), mainly due to an increased number of mild hypoglycaemias (1.0 [IQR 1.0–2.0] vs.1.0 [IQR 0.0–1.0], *p* < 0.001) (Fig. 1).

### Impact of AKI duration and severity on hypoglycaemia and mortality

**Fig. 2:**
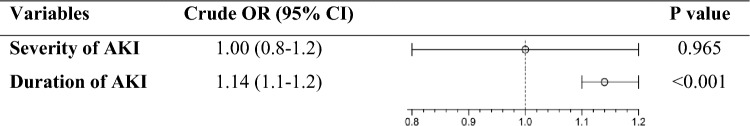
Impact of AKI severity and duration on hypoglycaemia. *CI* Confidence Interval, *OR* Odds Ratio

Patients in the AKI group had higher 30-day mortality (14.1% vs 7.3%, *p* = 0.019). AKI severity and duration were both associated with mortality, with 30-day mortality increasing through the stages of AKI (OR_stage 2/stage 1:_ 2.4, 95% CI: 1.0–59; OR_stage 3/stage 1_: 3.5, 95% CI: 1.3–9.4) and increasing by 16% for each additional day with AKI (95% CI: 1.1–1.2). AKI severity did not show a significant association with the risk of hypoglycaemia (Fig. 2). In contrast, each day of AKI duration was associated with a 14% increased risk of hypoglycaemia.

**Fig. 3:**
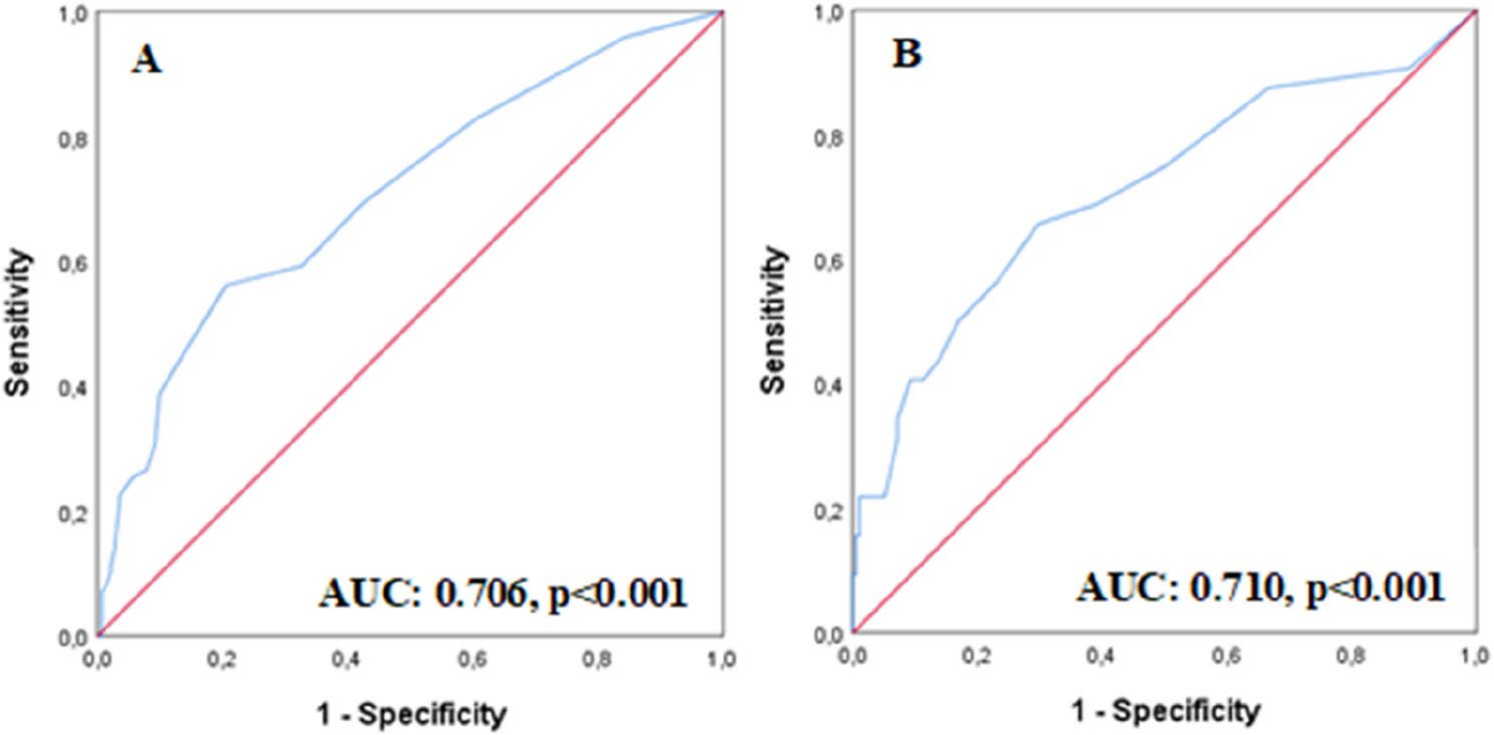
ROC Curves for AKI duration as predictor of hypoglycaemia (**A**) and mortality (**B**)

Furthermore, we found a cutoff of 5.5 days of AKI duration associated with increased risk of hypoglycaemia (sensitivity: 56.7%, specificity: 79.6%) and 30-day mortality (sensitivity: 65.6%, specificity: 70.3%) (Fig. 3).

### Hypoglycaemia, length of hospital stay and mortality

Globally, patients with hypoglycaemia had longer duration of hospital stay (13.0 days [IQR 9.0–21.0] vs 9.0 days [IQR 7.0–13.0], *p* < 0.001), with an average of 5.5 additional days of hospitalization (95% CI: 3.7–7.2, *p* < 0.001). Each additional hypoglycaemic event translated into an average of 3.0 additional days of hospitalization (95% CI: 2.5–3.5, *p* < 0.001). Hypoglycaemia was associated with higher 30-day mortality (22.3% vs 6.1%, *p* < 0.001). On univariate analysis, patients with hypoglycaemia had 4.4 times greater risk of death within 30 days (95%CI: 2.4–8.2, *p* < 0.001), and patients with at least one severe hypoglycaemia had 4.7 times greater risk (95% CI: 2.4–8.9, *p* < 0.001). Furthermore, each additional hypoglycaemic event increased the odd of death in 30 days by 42% (OR 1.42, 95% CI: 1.2–1.7, *p* < 0.001). On multivariate analysis, accounting for hypoglycaemia, AKI and age, hypoglycaemia was the main factor associated with increased risk of 30-day mortality (OR 3.6, 95% CI: 1.9–6.9, *p* < 0.001).

## Discussion

In this cohort study, we show that patients with type 2 diabetes who experienced AKI during hospitalization in medical wards had increased risk for hypoglycaemia during hospitalization. The prevalence of AKI during hospitalization of patients with diabetes was slightly higher than the one described for the general adult population [13]. Patients in the AKI group were older, had lower baseline eGFR, higher prevalence of hypertension and were more frequently admitted for infectious diseases. The prevalence of hypoglycaemia and the number of hypoglycaemic events per patient were higher in the AKI group, and the first hypoglycaemic event occurred with a median delay of 6 days after the detection of AKI. On multivariate analysis, the risk of hypoglycaemia was 4.2 times greater in the AKI group. The risk of hypoglycaemia increased with the duration of AKI, but was not significantly affected by AKI severity. Nonetheless, both AKI duration and severity were associated with mortality. We established a cutoff of 5.5 days of duration of AKI as a predictor of increased risk of hypoglycaemia and mortality. Additionally, hypoglycaemia during hospitalization was associated with longer duration of hospital stay and higher mortality, such as described in previous studies.

After analyzing previous literature, the main differences found between the two groups are comprehensible, since advanced age, CKD, hypertension and infectious disease are all known causes of increased susceptibility to AKI [14]. Comparing with the studies in the ICU setting, our results support the same association between AKI and hypoglycaemia in patients hospitalized in general medical wards, but with a smaller risk increase (4.2-fold vs tenfold). This might be due to less severe illness and less strict insulin protocols. Our results are also in line with the findings of Hung et al. [17] in several aspects: first, the fact that hypoglycaemia occurred with a substantial delay after AKI suggests that patients who are discharged soon after AKI may be at increased risk for hypoglycaemia after discharge; second, the fact that the duration of AKI had a significant impact on the risk of hypoglycaemia in our study is a possible explanation for what the authors described as a further increased risk of hypoglycaemia in patients with lesser degrees of renal recovery at discharge [17].

But our findings go beyond previous discoveries. By analyzing the impact of AKI characteristics on hypoglycaemia, we established that even mild elevations of serum creatinine, such as ≥ 0.3 mg/dL within 48 h, can harbor a significant risk of hypoglycaemia if they are prolonged over time, especially if they persist for more than 5 days. In clinical practice, these findings are extremely relevant, since AKI is highly prevalent during hospitalization and frequently underdiagnosed and undertreated when mild. Furthermore, patients are often discharged when renal function starts to improve. Those patients would probably benefit from insulin dose reduction in the post-discharge period and increased surveillance. During hospitalization, specific protocols to avoid hypoglycaemia and its burden in patients who experience AKI are also needed.

The main weaknesses of our study include its retrospective nature and the heterogeneity between groups. To minimize confounding and ensure that the two groups were comparable regarding the risk of hypoglycaemia, we used multiple logistic regression including all clinically relevant variables that differed between groups, using a significance level of *p* < 0.1. Still, and despite our strategies to reduce confounding factors, there may be residual confounders and, therefore, causality cannot be assumed. Simultaneously, the number of hypoglycaemic events and the duration of AKI might have been underestimated, given the fact that patients did not have continuous glucose monitoring systems and some did not have daily serum creatinine measurements. Strengths of our study include the fact that all cases of AKI were individually confirmed on the exposure group and excluded from the control group, based on AKI definition by KDIGO, and not only relying on the presence or absence of this diagnosis in clinical records. Daily capillary blood glucose records were analyzed, which implies that not only symptomatic hypoglycaemias were accounted for, as occurred in many other retrospective studies. Adding to this, extensive data were collected to identify possible confounding factors, such as insulin regimen during hospitalization and adjustments to outpatient insulin therapy on admission. Moreover, our study not only analyzed the risk of hypoglycaemia in hospitalized patients with AKI and type 2 diabetes, but also analyzed the impact of AKI characteristics on hypoglycaemia, which remained a gap in prior knowledge.

In conclusion, our results support AKI as an important risk factor for hypoglycaemia during hospitalization of patients with type 2 diabetes and its duration as the main factor increasing the risk of hypoglycaemia. These results pave the way for new approaches aiming to avoid hypoglycaemia in patients experiencing AKI during hospitalization.

## Data Availability

The datasets generated during and/or analyzed during the current study are available from the corresponding author on reasonable request.
